# Effects of Saliva From Periodontally Healthy and Diseased Subjects on Barrier Function and the Inflammatory Response in *in vitro* Models of the Oral Epithelium

**DOI:** 10.3389/froh.2021.815728

**Published:** 2022-01-05

**Authors:** Antoine Roy, Amel Ben Lagha, Reginaldo Gonçalves, Daniel Grenier

**Affiliations:** Oral Ecology Research Group, Faculty of Dentistry, Université Laval, Quebec City, QC, Canada

**Keywords:** epithelial barrier, epithelial cells, inflammatory mediators, matrix metalloproteinase, periodontitis, saliva

## Abstract

**Background:** Periodontitis is a multifactorial, bacteria-mediated chronic inflammatory disease that results in the progressive destruction of the tooth-supporting tissues. It is well-known that saliva from subjects suffering from this disease generally contains higher levels of pro-inflammatory mediators, matrix metalloproteinases (MMP), and bacteria-derived toxic products. The aim of this study was to investigate and compare the effects of saliva from periodontally healthy and diseased subjects on the barrier function and inflammatory response in *in vitro* models of the oral epithelium.

**Methods:** Unstimulated saliva samples from two groups of subjects, one with a healthy periodontium (*n* = 12) and one with severe generalized periodontitis (*n* = 11), were filter-sterilized. All the saliva samples were analyzed using an immunological multiplex assay to determine the levels of various cytokines and MMPs relevant to periodontitis. The impact of saliva on epithelial barrier integrity was assessed by monitoring transepithelial electrical resistance (TER) in an oral epithelium model using the B11 keratinocyte cell line. GMSM-K oral epithelial cells were treated with saliva from both groups to determine their ability to induce the secretion of interleukin-6 (IL-6) and interleukin-8 (IL-8), as determined by an enzyme-linked immunosorbent assay (ELISA).

**Results:** Saliva from the periodontitis subjects contained significantly higher concentrations of matrix metalloproteinase-8 (MMP-8), matrix metalloproteinase-9 (MMP-9), IL-8, and C-X-C motif chemokine ligand 1 (CXCL1) compared to saliva from the healthy subjects. Saliva from the healthy and periodontitis subjects affected cytokine secretion and TER in a similar manner. More specifically, saliva from both groups increased TER and induced IL-6 and IL-8 secretion in the *in vitro* oral epithelium models used.

**Conclusion:** Independently of the presence or absence of periodontitis, saliva can increase the relative TER and the secretion of IL-6 and IL-8 in *in vitro* models of the oral epithelium.

## Introduction

Periodontitis is a multifactorial, bacteria-mediated chronic inflammatory disease that results in the progressive destruction of the tooth-supporting tissues [1]. Data from epidemiological studies have shown that up to 46% of the adult population is affected by the disease, with different degrees of severity [2]. Periodontitis is primarily initiated by a dysbiotic dental biofilm that accumulates in the subgingival areas around the teeth, although disease progression is largely influenced by the host immuno-inflammatory response and a variety of risk factors such as smoking and uncontrolled diabetes [3, 4]. Over the past few decades, several Gram-negative anaerobic bacterial species have been clearly associated with the disease [5–7]. These periodontal pathogens produce a variety of toxins and proteolytic enzymes that can contribute to periodontal tissue destruction [8, 9]. Although these bacteria can directly mediate tissue breakdown, most of the destruction observed in periodontitis is caused by a chronic, exaggerated, and uncontrolled inflammatory response against them [3]. This chronic inflammatory state is characterized by the release of pro-inflammatory cytokines, which subsequently leads to matrix metalloproteinase (MMP) production, connective tissue damage, and bone resorption [10, 11].

Periodontal disease is associated with clinical signs of inflammation and an increase in gingival crevicular fluid (GCF) volume and flow [12]. GCF is an exudate from the wall of the gingival sulcus that exerts a protective effect against periodontal pathogens [13]. GCF from sites exhibiting periodontitis contains a variety of pro-inflammatory mediators, toxic bacterial by-products, and host- and bacteria-derived proteolytic enzymes [14–16]. Ultimately, GCF and its content are released into the oral cavity and mix with the whole saliva. Consequently, active periodontitis is associated with elevated salivary concentrations of pro-inflammatory mediators, including interleukin-1 beta (IL-1β), interleukin-6 (IL-6), interleukin-8 (IL-8), and tumor necrosis factor alpha (TNF-α) [17–21]. In addition, matrix metalloproteinase-8 (MMP-8), matrix metalloproteinase-9 (MMP-9), and matrix metalloproteinase-13 (MMP-13) are present in higher levels in the saliva of individuals with periodontitis than in the saliva of healthy individuals [18–21]. The above host-derived factors have been proposed as salivary biomarkers for the diagnosis of periodontitis [17–21].

The effect of human saliva on host cells has been assessed in various studies. It has been reported that the exposure of mouse-derived macrophages to human saliva leads to cell differentiation into a pro-inflammatory phenotype and, consequently, increased IL-6 and IL-12 production [22]. Likewise, three pro-inflammatory cytokines, namely IL-8, C-X-C motif chemokine ligand 1 (CXCL1), and C-X-C motif chemokine ligand 2 (CXCL2), that are produced by human gingival fibroblasts are up-regulated following exposure to saliva [23]. Moreover, gingival fibroblasts express these same chemokines when exposed to artificial saliva containing bovine mucins [24]. On the other hand, human oral epithelial cells from the HSC-2 cell line do not exhibit a similar inflammatory response when exposed to human or artificial saliva [23, 24]. Cvikl et al. also reported that saliva is a potent inducer of IL-6 and IL-8 production by oral fibroblasts but not by oral keratinocytes [25]. The exposure of oral keratinocytes to salivary leptin isolated from human saliva increases their proliferation and induces the secretion of keratinocyte growth factor (KGF) and EGF [26]. To the best of our knowledge, there is no current information available regarding the effect of saliva from periodontally diseased subjects on epithelial barrier integrity and the inflammatory response. The purpose of the present study was to investigate and compare the effects of saliva from healthy and periodontitis subjects on epithelial barrier integrity and the inflammatory response in *in vitro* models of the oral epithelium.

## Materials and Methods

### Subject Recruitment

A group of subjects with a healthy gingiva (*n* = 12) and a group of subjects with severe generalized periodontitis (*n* = 11) were recruited for the study from among the patients, staff, and students of the Faculty of Dentistry at Université Laval. The recruitment and saliva sampling were authorized by the local Health Research Ethics Board (File number 2018-291A-1/18-03-2019). All the participants provided written informed consent and were admitted to the study following a complete periodontal examination. The participants in the healthy group had to display a bleeding on probing score <10%, probing depths ≤ 3 mm, and no periodontal attachment loss due to periodontal disease. The subjects in the periodontitis group had to exhibit generalized stage III or IV periodontitis, grade B or C, according to the most recent classification of periodontal disease [27]. Subjects were excluded based on the following criteria: (i) being <18 years old, (ii) having fewer than 20 teeth remaining, (iii) having received a periodontal treatment in the previous 12 months, (iv) being a smoker, (v) having used antibiotics and/or immunosuppressive drugs in the previous 3 months, and (vi) presenting a systemic disease that affects the immune system or periodontal health.

### Saliva Sampling and Processing

Saliva collection was based on the procedures outlined by Navazesh and Kumar [28]. Unstimulated saliva was obtained from the subjects, who were asked not to eat, chew gum, or use any dental hygiene products for at least 1 hour before the saliva was collected. Approximately 5 ml of saliva was collected, over a period of 10–15 min, in sterile polypropylene tubes from each subject. The samples were kept on ice until being centrifuged at 15,000 *g* for 20 min. Saliva samples were sterilized by filtration through a 0.2-μm pore size membrane filter, and stored at −80°C until used.

### Analysis of Saliva Samples for Cytokines and MMPs

The saliva samples were analyzed to determine their cytokine and MMP content. Two 50-μL aliquots of each saliva sample were sent to Eve Technologies (Calgary, AB, Canada) for analysis by multiplexing technology using a bead analyzer, which includes a dual laser system and a flow cytometry system. Multiplex assays for cytokines (Human Cytokine 65-Plex Discovery Assay) and MMPs (Human MMP 9-Plex, TIMP 4-Plex Discovery Assay) were performed.

### Effect of Saliva on Epithelial Barrier Integrity

The effect of saliva on the barrier function of the oral epithelium was assessed in an *in vitro* model using the human oral keratinocyte B11 cell line [29], which was kindly provided by Dr. S. Groeger (Justus-Liebig-University of Giessen, Giessen, Germany). This cell line was isolated from a biopsy of the buccal gingiva and immortalized with a combination of the human papilloma virus onkoproteins E6 and E7 [29]. Epithelial barrier integrity was evaluated by monitoring transepithelial electrical resistance (TER) using the protocol previously described by Ben Lagha and Grenier [30]. The keratinocytes were cultivated at 37°C in a 5% CO_2_ atmosphere in Keratinocyte Serum-Free Medium (K-SFM) supplemented with bovine pituitary extract (50 μg/ml), recombinant human epidermal growth factor (5 ng/ml), and penicillin G-streptomycin (100 μg/ml). The cells were seeded into a Costar® 6.5 mm Transwell® with 10-μm thick and 4-μm pore polyester membrane inserts (Corning Co., Cambridge, MA, USA) at a concentration of 3.5 x 10^5^ cells per insert. The inserts were incubated for 72 h to allow tight junctions to form between the cells. Saliva samples were diluted 50, 25, 12.5, and 6.25% (v/v) in K-SFM and were added (100 μl) to the apical compartment. K-SFM alone was used for the negative control. Resistance between the apical and basal compartments was determined in triplicate using a manual epithelial Ohm-meter (EVOM2; World Precision Instruments, Sarasota, FL, USA) before and after 3, 6, 24, 48, and 72 h of exposure to each dilution of the individual saliva samples. The mean ± standard deviation (SD) of the TER values of each subject groups was calculated, and the relative TER was then expressed as a percentage of the measurements recorded prior to the addition of the saliva samples.

### Effect of Saliva on the Inflammatory Response of Oral Epithelial Cells

The effect of saliva on IL-6 and IL-8 secretion by oral epithelial cells was assessed with an *in vitro* model using the GMSM-K cell line [31], which was kindly provided by Dr. V. Murrah (University of North Carolina, Chapel Hill, NC, USA*)*, according to a previously described protocol [32]. These oral epithelial cells were isolated from a stillborn male fetus and transfected with the shuttle vector plasmid, pZ189, containing the T-antigen-coding region and replication origin from the Simian virus 40 (SV40) [31]. This cell line has an epithelial phenotype, which was verified by electron microscopic and immunohistochemical analyses [31]. GMSM-K cells were cultivated at 37°C in a 5% CO_2_ atmosphere in Dulbecco's modified Eagle's medium (DMEM) supplemented with 10% heat-inactivated fetal bovine serum (FBS) and penicillin G-streptomycin (100 μg/ml). The epithelial cells, at a concentration of 1 x 10^6^ cells/ml, were seeded (100 μl) into wells of a 96-well microplate and incubated for 24 h to allow cell adhesion. Thereafter, cells were washed with DMEM (lacking FBS and antibiotics) prior to add saliva samples, diluted 50, 25, 12.5, and 6.25% in DMEM (100 μl; lacking FBS and antibiotics) to the wells in triplicate assays. DMEM alone was used for the negative control. The cells were then incubated (37°C/5% CO_2_) for an additional 24 h before collecting the cell-free supernatants. The supernatant samples were stored at −20°C until IL-6 and IL-8 concentrations were determined using commercial enzyme-linked immunosorbent assay (ELISA) kits (R&D Systems, Minneapolis, MN, USA) according to the manufacturer's protocol. The net amount of IL-6 and IL-8 secreted by the cells was determined by subtracting the initial concentrations of these cytokines in each saliva sample, which were determined by ELISA. Assays were performed in triplicate in two independent experiments and the means ± SD were calculated.

The effect of the stimulation described above on the viability of GMSM-K epithelial cells was assessed using a MTT 3-(4,5-dimethylthiazol-2-yl)-2,5-diphenyltetrazolium bromide) (MTT) colorimetric assay according to the manufacturer's protocol (Roche Diagnostics GmbH, Mannheim, Germany). The assays were performed in triplicate, and the relative viability was calculated. A value of 100% viability was assigned to control cells treated with DMEM alone.

### Statistical Analysis

An unpaired student's t-test was performed to compare the composition of the saliva samples from both groups. The effect of the saliva samples on epithelial cells was determined by comparing the means of both groups and their controls using a one-way ANOVA followed by Bonferroni multiple comparison *post hoc* test. A *p* < 0.05 was considered statistically significant. The statistical analysis was performed using Prism 8 (GraphPad Software Inc, San-Diego, USA).

## Results

**Figure 1:**
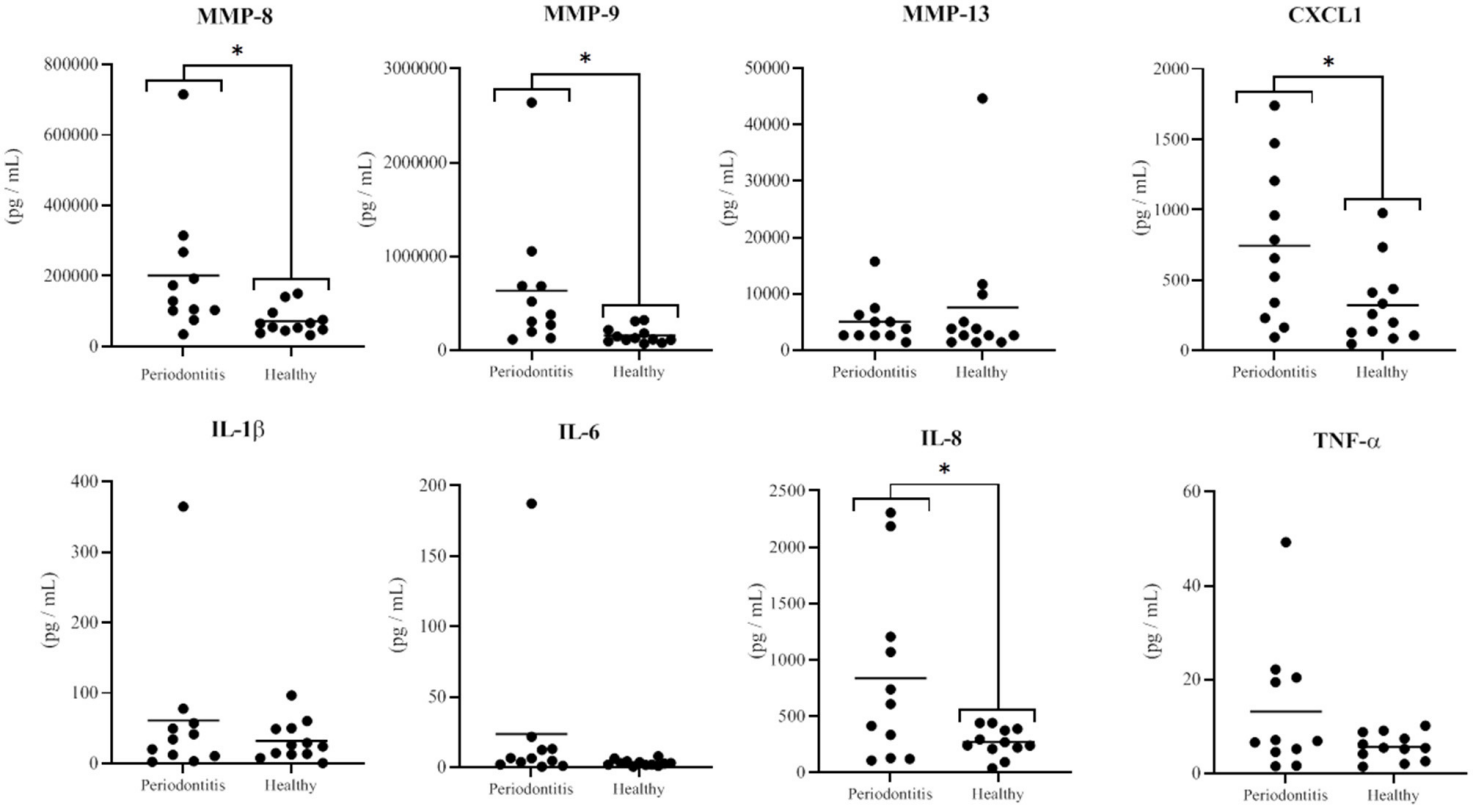
Concentrations of MMPs and cytokines in saliva samples from the periodontitis and healthy subjects. The horizontal black lines represent the mean values. The differences between the mean salivary levels of the two groups were considered significant at *p* < 0.05 (*).

The saliva samples from the healthy and periodontitis subjects were analyzed using multiplexing technology in order to compare their content in various MMPs, TIMPs, and cytokines. Saliva from the periodontitis subjects had significantly higher mean levels of MMP-8 (*p* = 0.03) and MMP-9 (*p* = 0.03) than saliva from the healthy subjects (Figure 1). The mean concentrations of MMP-8 and MMP-9 in the saliva from the periodontitis subjects were, respectively, 2.8 and 4.0 times higher than those in the saliva from the healthy subjects. The mean salivary concentrations of CXCL1 and IL-8 were 2.3 times higher (*p* = 0.03) and 3.1 times higher (*p* = 0.02), respectively, in the periodontitis subjects than in the healthy subjects (Figure 1). The concentrations of other biomarkers that have previously been reported to be higher in the saliva of periodontitis patients, including IL-1β, IL-6, TNF-α, and MMP-13 [17–21], were not detected in significantly higher concentrations in the saliva of the periodontitis subjects than in the saliva of the healthy subjects (Figure 1). All the additional cytokines, MMPs, and TIMPs included in the multiplex assays were either found in similar amounts in saliva samples of the two subject groups or detected in negligible amounts.

**Figure 2:**
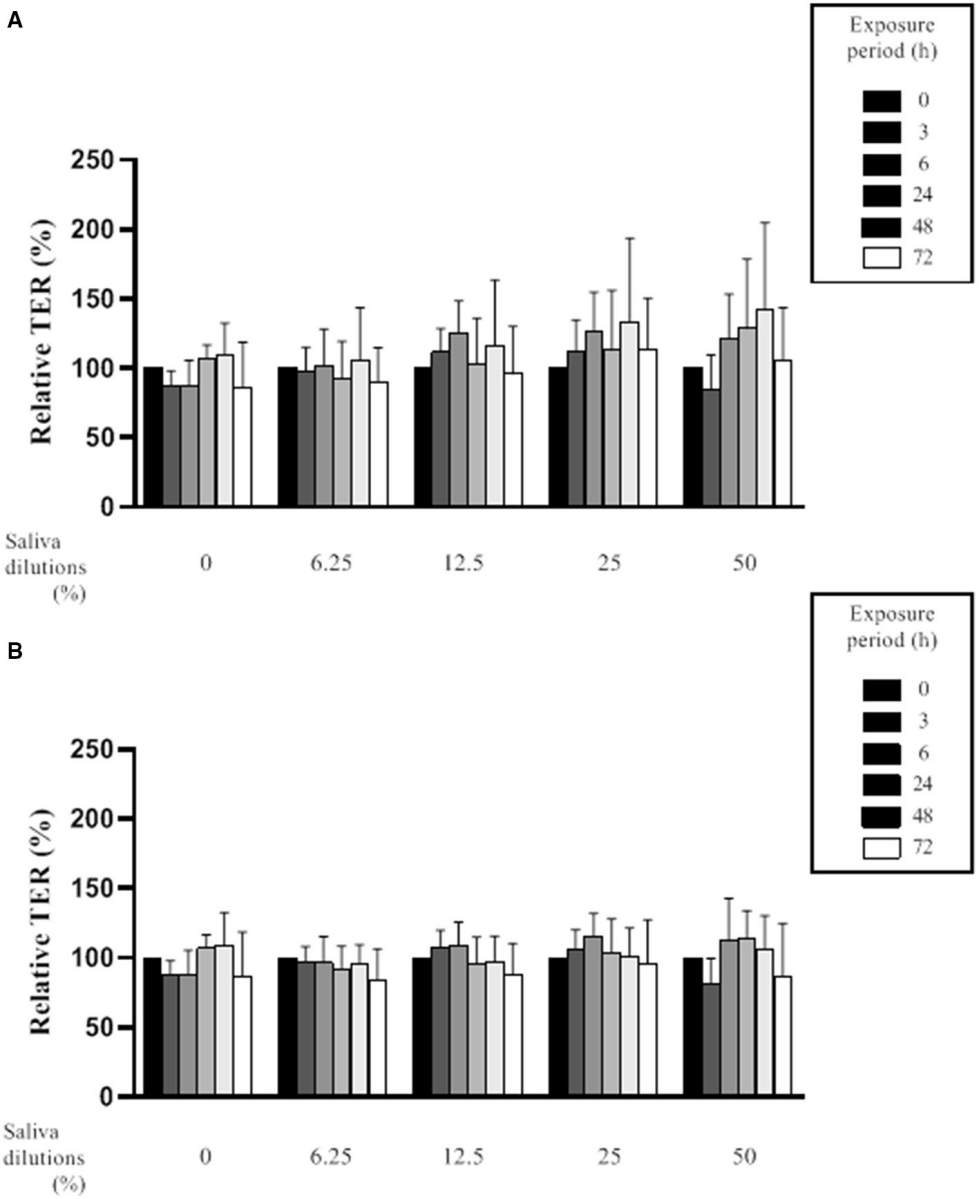
Effect of various dilutions of filter-sterilized saliva from periodontitis subjects **(A)** and healthy subjects **(B)** on the relative transepithelial electrical resistance (TER) of an epithelial barrier model following different exposure periods. Data are expressed by group (periodontitis and healthy) as the mean ± SD of the relative TER determined in triplicate assays. No significant differences (*p* < 0.05) were observed between the periodontitis and healthy subjects at any saliva dilution or time point.

The effect of saliva on the integrity of the epithelial barrier was evaluated by assessing variations in TER values in an *in vitro* model of oral keratinocytes (B11 cell line) following exposure to saliva samples from both groups of subjects. The statistical analysis revealed no significant differences in the mean ± SD of the relative TER for the *in vitro* model exposed to saliva samples from periodontitis or healthy subjects at any dilution (6.25, 12.5, 25, and 50%) or time point (0, 3, 6, 24, 48, and 72 h) tested (Figure 2). Although saliva (50% dilution) from the periodontitis subjects showed a tendency to increase TER following a 24- or 48-h exposure, this was not significant.

**Figure 3:**
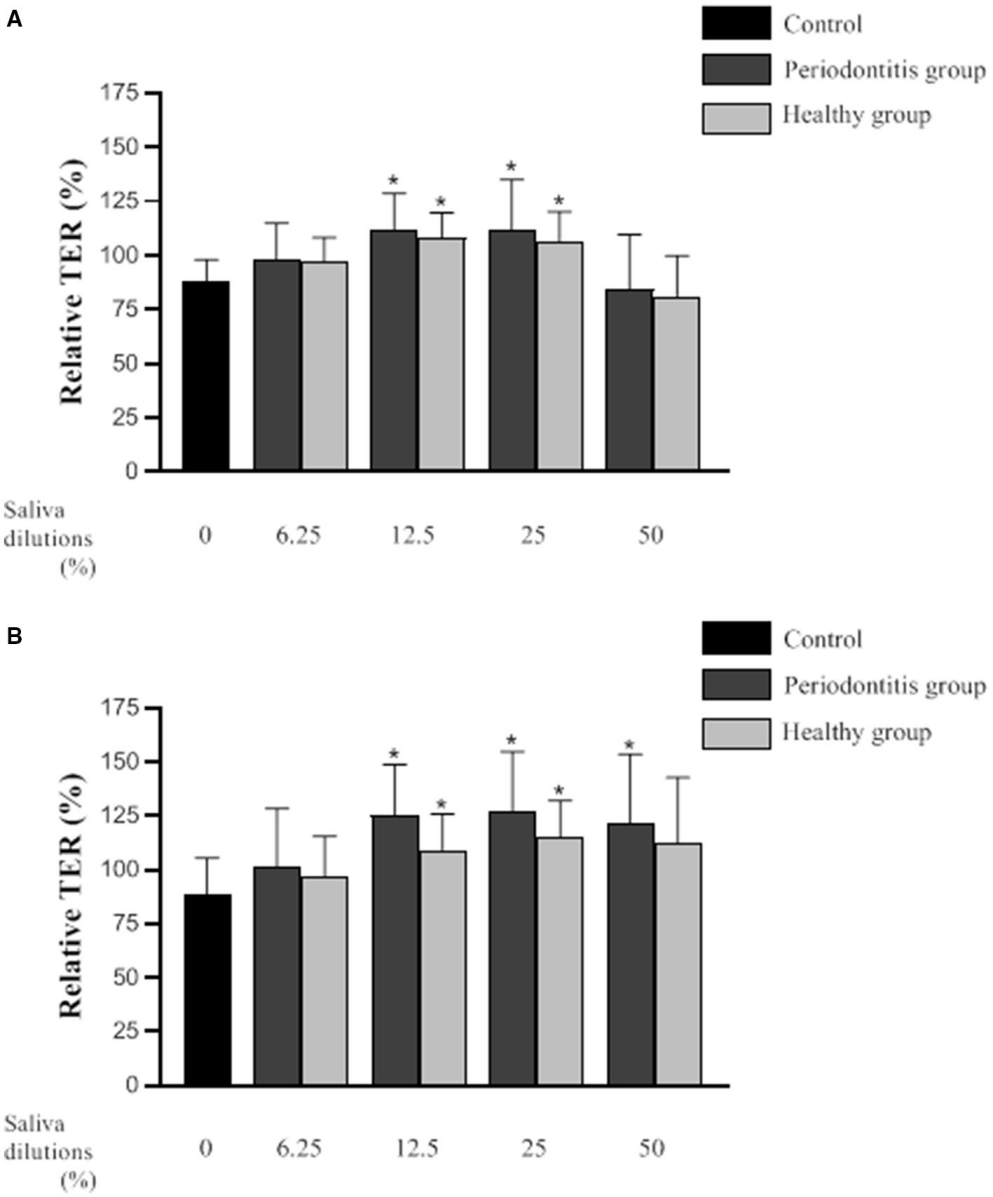
Effect of various dilutions of filter-sterilized saliva from the periodontitis and healthy subjects on the relative transepithelial electrical resistance (TER) of an epithelial barrier model following a 3-h **(A)** and 6-h **(B)** exposure. Data are expressed by group as the mean ± SD of the relative TER determined in triplicate assays. A difference with the control group was considered significant at *p* < 0.05 (*). No significant difference*s* (*p* < 0.05) were observed between the periodontitis and health*y* subjects at any saliva dilution or time point.

Figure 3 shows the relative TER values (mean ± SD) of the *in vitro* oral epithelium model following 3- and 6-h exposures to saliva from the periodontitis and healthy subjects. Compared to the controls (culture medium instead of saliva), the TER values were significantly higher following a 3-h exposure to saliva (12.5 and 25% dilutions) from either group. More specifically, a treatment of the *in vitro* model with saliva (25% dilution) from the periodontitis and healthy subjects was associated with TER values that were 18 and 24% higher than the controls, respectively. Conversely, a 3-h exposure to saliva (50% dilution) from either group caused a decrease in TER values relative to the controls. However, the difference was not statistically significant.

A 6-h exposure to saliva (12.5 and 25% dilutions) from either the periodontitis or the healthy subjects resulted in significantly higher TER values than those of the control (Figure 3). More specifically, the TER values were 27 and 38% higher than that of the control for the *in vitro* oral epithelium model treated with saliva (25% dilution) from the periodontitis or healthy subjects, respectively. Following a 6-h treatment with saliva (50% dilution), TER was significantly higher than that of the control only for the *in vitro* model treated with saliva from the periodontitis subjects.

**Figure 4:**
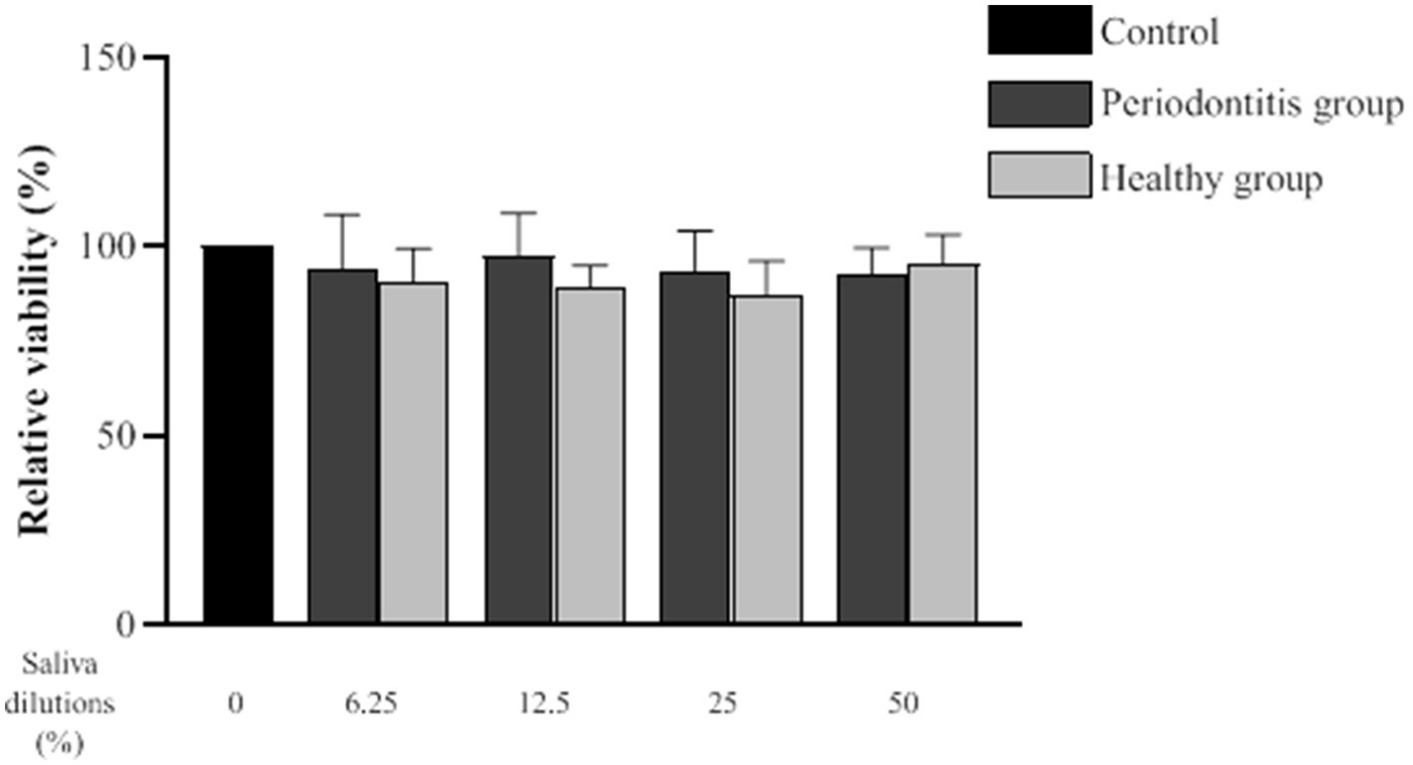
Effect of various dilutions of filter-sterilized saliva from the periodontitis and healthy groups on epithelial cell viability. The MTT colorimetric assay was performed after a 24-h exposure to saliva. Data are expressed by subject group as the mean ± SD of the relative cell viability values from triplicate assays. No significant difference (*p* < 0.05) was observed between the periodontitis, healthy, and control groups at any saliva dilution.

The effect of saliva from the periodontitis and healthy subjects on the epithelial inflammatory response was investigated using human oral epithelial cells (GMSM-K cell line). To exclude the possibility that a decreased inflammatory response by the epithelial cells may be caused by a cytotoxic effect, we evaluated the impact of saliva from both groups on cell viability using a MTT colorimetric assay. Epithelial cell viability following a 24-h exposure to the saliva samples is shown in Figure 4. A statistical analysis showed that there is no difference in epithelial cell viability between the periodontitis and healthy subjects or the negative control.

**Figure 5:**
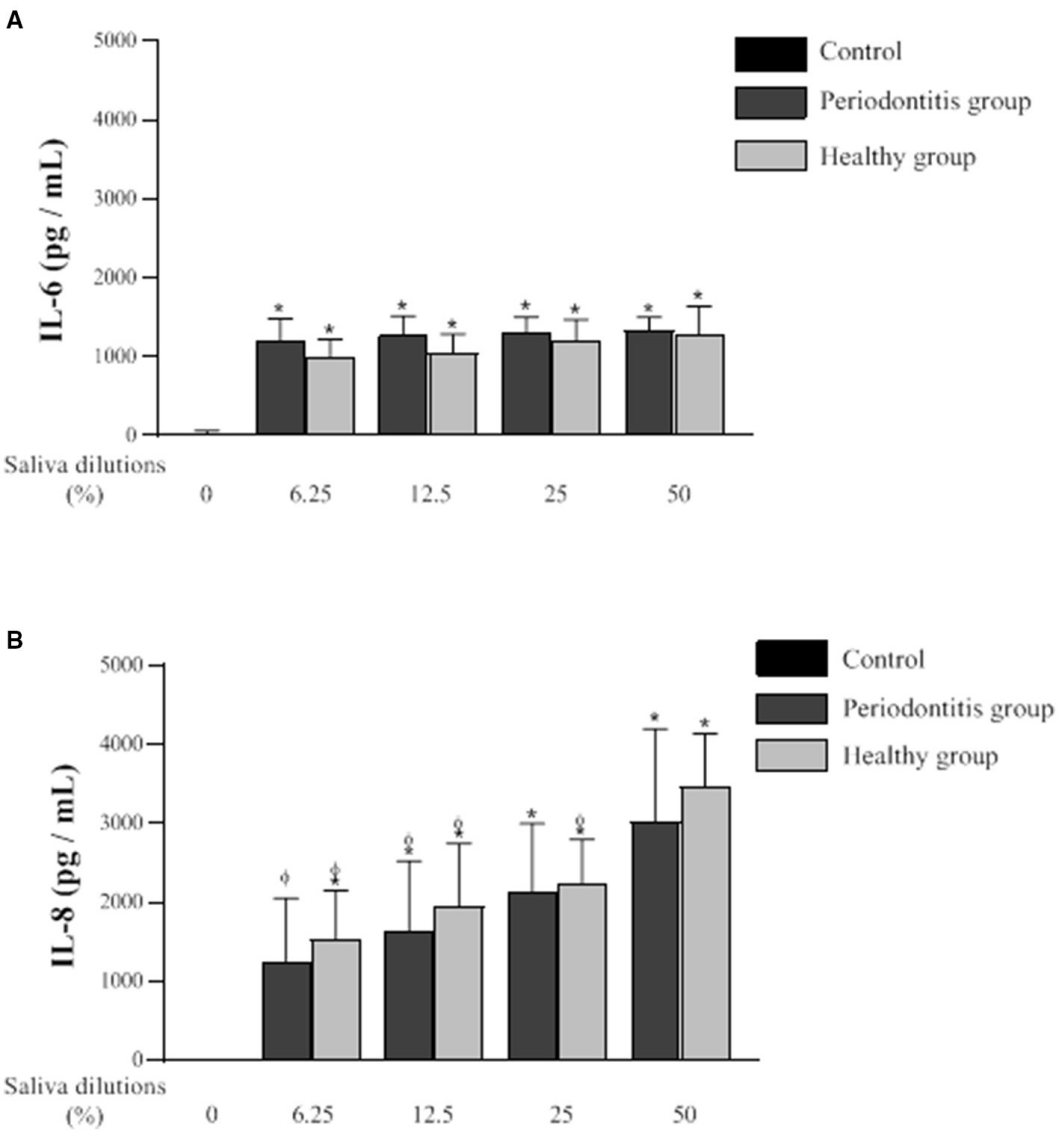
Effect of various dilutions of filter-sterilized saliva from the periodontitis and healthy subjects on the secretion of IL-6 **(A)** and IL-8 **(B)** by oral epithelial cells (GMSM-K cell line) following a 24-h exposure. Data are expressed by subject group as the mean ± SD of cytokine concentrations determined in triplicate assays. The difference with the control group was considered to be significant at *p* < 0.05 (*). A difference with the respective 50% saliva dilutions was considered to be significant at *p* < 0.05 (ϕ). No significant difference was observed between the periodontitis and healthy groups at any saliva dilution.

The secretion of IL-6 and IL-8 by oral epithelial cells treated (24 h) with saliva samples from the two groups at various dilutions is shown in Figure 5. The net amount of IL-6 and IL-8 secreted by the cells was determined by subtracting the levels of the cytokines in each saliva sample as determined by ELISA. Compared to the controls, the saliva samples from both groups induced significant IL-6 secretion by the epithelial cells. However, no significant difference in saliva-induced IL-6 secretion by the periodontitis and healthy subjects was observed. IL-6 concentrations of 1285.7 pg/ml and 1332.2 pg/ml were detected, respectively, following exposure to saliva (50% dilution) from the periodontitis and healthy subjects. The saliva dilutions tested (50, 25, 12.5, and 6.25%) did not give rise to any significant differences with respect to the amounts of IL-6 secreted by the epithelial cells.

Oral epithelial cells stimulated with saliva from one or the other of the two subject groups secreted significantly higher amounts of IL-8 compared to the control (Figure 5). The difference in IL-8 secretion by epithelial cells in response to the saliva from each group was not statistically significant. However, unlike IL-6, the amounts of IL-8 secreted by epithelial cells after the exposure to saliva were influenced by the dilution of the saliva samples. IL-8 secretion was significantly higher when the saliva samples from the two groups were used at their lowest dilution (50%). The treatment of epithelial cells with saliva from the healthy subjects induced a significantly higher secretion of IL-8 at a dilution of 50% compared to saliva at dilutions of 25, 12.5, and 6.25%. The saliva from the periodontitis subjects at a 50% dilution also induced a significantly higher secretion of IL-8 compared to saliva at dilutions of 12.5 and 6.25%. The treatment of the oral epithelial cells with saliva (50% dilution) from the periodontitis and healthy subjects resulted in IL-8 levels of 3465.4 and 3017.1 pg/ml, respectively.

## Discussion

Periodontitis is a chronic inflammatory disease that causes the progressive destruction of the tooth-supporting structures, including the alveolar bone. Although the disease has been associated with the presence of a number of Gram-negative anaerobic bacterial species in the subgingival dental biofilm, most of the damage caused to periodontal tissues is modulated by the host immuno-inflammatory response [3, 7]. A dysregulated inflammatory reaction by the host against local microorganisms has been observed during periodontitis and is characterized by a production of potent pro-inflammatory mediators such as IL-1β, TNF-α, IL-6, and IL-8, as well as various protein-degrading enzymes such as MMP-8, MMP-9, and MMP-13, leading to localized destruction [33, 34]. These host-derived factors, along with various toxic bacterial by-products, can be found in the GCF, which is a fluid expelled from the gingival sulcus and ultimately mixes with the whole saliva in the oral cavity [17–21]. Consequently, various markers, indicative of periodontitis, can be found in saliva [17–21]. In the present study, saliva samples from periodontitis and healthy subjects were compared with respect to their cytokine and MMP content as well as their effects on barrier function and the inflammatory response in *in vitro* models of the oral epithelium.

In this study, saliva, instead of GCF, was used since it offers several advantages, being produced in much greater amounts (required for assays) and not requiring an invasive procedure. Unstimulated saliva samples obtained from the subjects with severe generalized periodontitis were analyzed by multiplexing for their content of various MMPs and inflammatory mediators and were compared to the results from saliva samples collected from a second group of periodontally healthy subjects. This analysis revealed that the levels of salivary MMP-8 and MMP-9 were significantly higher in the periodontitis subjects. This observation is in agreement with previous studies that investigated salivary markers for periodontitis. Ramseier et al., for example, compared biomarkers in unstimulated whole saliva from subjects with gingivitis and from periodontally healthy subjects to subjects with mild to severe periodontitis [35]. They reported significantly elevated levels of MMP-8 and MMP-9 in saliva from the periodontitis subjects compared with saliva from the healthy subjects [35]. Ebersole et al. also reported higher salivary MMP-8 levels in subjects with periodontitis compared to subjects with gingivitis or a healthy periodontium [36].

In the present study, we also observed that the levels of IL-8 and CXCL1, two neutrophil chemoattractant chemokines, were significantly higher in the saliva from the periodontitis subjects than in the saliva from the healthy subjects. These two salivary biomarkers are not usually reported as being present at higher levels in periodontitis subjects [18, 20]. For instance, a similar study by Kawamoto et al. [37] compared various chemokine and cytokine levels in whole saliva from subjects with generalized severe periodontitis and from subjects with a healthy periodontium. Although their general method for patient selection and saliva sampling was comparable to that of the present study, no significant difference was observed for CXCL1 and IL-8 levels [37]. Ramseier et al. [35] also reported no significant differences between the two subject groups with respect to IL-1β, IL-6, and TNF-α levels, which is in agreement with our results. However, these observations are not consistent with other studies that reported elevated salivary IL-1β and IL-6 levels in periodontitis subjects. Ebersole et al. [36] evaluated IL-1β, and IL-6 concentrations in saliva from a cohort of 209 subjects with periodontitis, gingivitis, and a healthy periodontium and found that salivary levels of these biomarkers were more elevated in the periodontitis subjects than in the other two groups. According to our findings, while IL-1β and IL-6 levels had a tendency to be higher in the periodontitis group, the difference with the healthy group was not significant. These discrepancies can be explained in part by the small number of participants included in our study, as well as the saliva method collection and the cytokine quantification assay used.

The oral mucosa, which is composed of a layer of epithelial cells covering a fibrous connective tissue, separates and protects the underlying structures from the external environment. Epithelial cells are tightly connected to each other by a variety of transmembrane proteins to form the epithelial barrier, which is the first line of defense against periodontal pathogens and their toxins [38, 39]. Epithelial cells also play a major role in the activation of the inflammatory response in periodontal tissues by producing various cytokines, including IL-6 and IL-8 [38]. We investigated the effects of saliva from periodontally healthy subjects and from subjects with severe generalized periodontitis on oral epithelial cells (barrier function and cytokine secretion) and compared the two subject groups. To the best of our knowledge, there is no report in the scientific literature regarding the effect of saliva from periodontitis subjects on epithelial barrier integrity and the inflammatory response. The impact of saliva on epithelial barrier integrity was assessed by measuring TER in an *in vitro* model exposed to various dilutions of saliva. Saliva from the two groups of subjects modulated epithelial barrier function in a similar way since no difference was observed between the two groups. A significant increase in TER was observed following a short exposure (3 and 6 h) to the 25 and 12.5% dilutions of the saliva samples. In comparison, although not statistically significant, the 50% dilution of the saliva samples initially caused a slight decrease in TER after 3 h, which subsequently increased to values comparable to those observed for the *in vitro* models exposed for 6 h to higher saliva dilutions. Overall, these results suggested that saliva tends to promote a higher TER. This observation can be attributed to the presence of various growth factors in saliva such as EGF, or hormones like leptin, that can have a positive influence on epithelial cell migration and proliferation [26, 40]. Interestingly, no partial breakdown of the epithelial barrier, as indicated by a decrease in TER, was observed following the exposure to saliva collected from the periodontitis subjects. This suggests that bacterial by-products such as toxins and proteolytic enzymes in the saliva from periodontitis subjects were not present in sufficient amounts to negatively affect epithelial barrier integrity. MMP-9, which was found in higher concentration in saliva from periodontitis subjects in our study, has been reported to induce the breakdown of the intestinal epithelial barrier in a mouse model [41]. Since no impairment of epithelial barrier integrity was observed with the saliva samples from the periodontitis patients, this suggests that MMP-9 concentrations in the samples were either too low to have an effect or that the MMP-9 was in an inactive form.

In the present study, IL-6 and IL-8 secretion by oral epithelial cells (GMSM-K cell line) was assessed following the exposure to filter-sterilized saliva from periodontitis or healthy subjects. Significant secretion of IL-6 and IL-8 by epithelial cells was detected after a 24-h exposure to saliva from the two groups of subjects compared to the control. While the amount of IL-8 secreted by the cells decreased with an increase in the saliva dilution, no such effect was observed for IL-6. Pro-inflammatory mediators or bacterial by-products in the saliva from the two groups may have been responsible for inducing IL-6 and IL-8 secretion by the oral epithelial cells. It is also possible that the inflammatory response triggered by the saliva may in part be modulated by other substances that are constitutively present in saliva. Interestingly, salivary lactoferrin, an antibacterial cationic peptide, has been reported to induce the production of IL-8, IL-6, and MCP-1 by oral epithelial cells [42]. A study by Cvikl et al. [25] reported that IL-6 and IL-8 secretion by gingival fibroblasts, primary gingival epithelial cells, and immortalized oral epithelial cells (HSC-2 cell line) following a 24-h exposure to sterile saliva (10-fold dilution) from healthy donors induced a strong production of IL-6 and IL-8 by fibroblasts and that the epithelial cells secreted insignificant amounts of the two cytokines. These contradictory results may be explained by the fact that the saliva samples used in the present study were less diluted. Also, in the present study, the saliva samples were tested on GMSM-K epithelial cells, which may react differently than other cell lines. It is worth mentioning the study of Müller et al., who reported that a commercial artificial saliva (Glandosane®, Cell Pharm, Bad Vilbel, Germany) significantly increased IL-8 expression by oral epithelial cells (HSC-2 cell line) while the three other artificial saliva they tested did not have a substantial impact [24]. This indicates that small variations in the composition of artificial or natural saliva likely influence the inflammatory response of epithelial cells. The composition of saliva obtained following masticatory stimulation can vary slightly compared to unstimulated saliva [43]. It is thus also possible that the saliva collection protocol used in the present study may have caused the contradictory results that we and Müller et al. [24] obtained.

In conclusion, our study showed that filter-sterilized saliva collected from periodontitis subjects and from healthy subjects had a similar effect on the barrier function and the inflammatory response of *in vitro* oral epithelium models. Exposure to saliva generally tended to increase the TER of oral epithelial cells. This may help preventing periodontal pathogens to translocate through the epithelial barrier and reach the underlying connective tissue. In addition, we observed a significant increase in the secretion of IL-6 and IL-8 when the epithelial cells were exposed to the saliva samples. Within the limitations of the *in vitro* model used, this suggests that saliva may contribute to maintain an inflammarory state.

## Data Availability Statement

The original contributions presented in the study are included in the article/supplementary material, further inquiries can be directed to the corresponding author/s.

## Ethics Statement

The studies involving human participants were reviewed and approved by the Local Health Research Ethics Board (File number 2018 291A 1/18 03 2019) of Université Laval. The patients/participants provided their written informed consent to participate in this study.

## Author Contributions

DG and RG conceived and designed the experiments. AR and AB performed the experimental assays and the statistical analysis. AR drafted the manuscript. DG and RG reviewed and edited the manuscript. All authors contributed to the article and approved the submitted version.

## Funding

This study was supported by the Fonds Émile-Beaulieu.

## Conflict of Interest

The authors declare that the research was conducted in the absence of any commercial or financial relationships that could be construed as a potential conflict of interest.

## Publisher's Note

All claims expressed in this article are solely those of the authors and do not necessarily represent those of their affiliated organizations, or those of the publisher, the editors and the reviewers. Any product that may be evaluated in this article, or claim that may be made by its manufacturer, is not guaranteed or endorsed by the publisher.

## References

[1] Papapanou PN Sanz M Buduneli N Dietrich T Feres M Fine DH. Periodontitis: consensus report of workgroup 2 of the 2017 world workshop on the classification of periodontal and peri-implant diseases and conditions. J Periodontol. (2018) 89:173–82. 10.1002/JPER.17-0721 29926951

[2] Eke PI Dye BA Wei L Slade GD Thornton-Evans GO Borgnakke WS. Update on prevalence of periodontitis in adults in the United States: NHANES 2009 to 2012. J Periodontol. (2015) 86:611–22. 10.1902/jop.2015.140520 25688694 PMC4460825

[3] Meyle J Chapple I. Molecular aspects of the pathogenesis of periodontitis. Periodontol 2000. (2015) 69:7–17. 10.1111/prd.12104 26252398

[4] Van Dyke TE Sheilesh D. Risk factors for periodontitis. J Int Acad Periodontol. (2005) 7:3–7. 15736889 PMC1351013

[5] Socransky SS Haffajee AD Cugini MA Smith C Kent RL. Microbial complexes in subgingival plaque. J Clin Periodontol. (1998) 25:134–44. 10.1111/j.1600-051X.1998.tb02419.x 9495612

[6] Curtis MA Diaz PI Van Dyke TE. The role of the microbiota in periodontal disease. Periodontol 2000. (2020) 83:14–25. 10.1111/prd.12296 32385883

[7] Lamont RJ Koo H Hajishengallis G. The oral microbiota: dynamic communities and host interactions. Nature Rev Microbiol. (2018) 16:745–59. 10.1038/s41579-018-0089-x 30301974 PMC6278837

[8] O'Brien-Simpson NM Veith PD Dashper SG Reynolds EC. Antigens of bacteria associated with periodontitis. Periodontol 2000. (2004) 35:101–34. 10.1111/j.0906-6713.2004.003559.x 15107060

[9] Dahlen G Basic A Bylund J. Importance of virulence factors for the persistence of oral bacteria in the inflamed gingival crevice and in the pathogenesis of periodontal disease. J Clin Med. (2019) 8:1339. 10.3390/jcm8091339 31470579 PMC6780532

[10] Hajishengallis G Korostoff JM. Revisiting the Page & Schroeder model: the good, the bad and the unknowns in the periodontal host response 40 years later. Periodontol 2000. (2017) 75:116–51. 10.1111/prd.12181 28758305 PMC5539911

[11] Loos BG Van Dyke TE. The role of inflammation and genetics in periodontal disease. Periodontol 2000. (2020) 83:26–39. 10.1111/prd.12297 32385877 PMC7319430

[12] Griffiths GS. Formation, collection and significance of gingival crevice fluid. Periodontol 2000. (2003) 31:32–42. 10.1034/j.1600-0757.2003.03103.x 12656994

[13] Alfano MC. The origin of gingival fluid. J Theor Biol. (1974) 47:127–36. 10.1016/0022-5193(74)90103-9 4617812

[14] Barros SP Williams R Offenbacher S Morelli T. Gingival crevicular fluid as a source of biomarkers for periodontitis. Periodontol 2000. (2016) 70:53–64. 10.1111/prd.12107 26662482 PMC4911175

[15] Almehmadi AH Alghamdi F. Biomarkers of alveolar bone resorption in gingival crevicular fluid: a systematic review. Arch Oral Biol. (2018) 93:12–21. 10.1016/j.archoralbio.2018.05.004 29800801

[16] Arias-Bujanda N Regueira-Iglesias A Balsa-Castro C Nibali L Donos N Tomás I. Accuracy of single molecular biomarkers in gingival crevicular fluid for the diagnosis of periodontitis: a systematic review and meta-analysis. J Clin Periodontol. (2019) 46:1166–82. 10.1111/jcpe.13188 31444912

[17] Jaedicke KM Preshaw PM Taylor JJ. Salivary cytokines as biomarkers of periodontal diseases. Periodontol 2000. (2016) 70:164–83. 10.1111/prd.12117 26662489

[18] Arias-Bujanda N Arias-Bujanda N Regueira-Iglesias A Balsa-Castro C Nibali L Donos N. Accuracy of single molecular biomarkers in saliva for the diagnosis of periodontitis: a systematic review and meta-analysis. J Clin Periodontol. (2020) 47:2–18. 10.1111/jcpe.13202 31560804

[19] Sukriti KC Wang XZ Gallagher JE. Diagnostic sensitivity and specificity of host-derived salivary biomarkers in periodontal disease amongst adults: systematic review. J Clin Periodontol. (2020) 47:289–308. 10.1111/jcpe.13218 31701554

[20] Zhang Y Kang N Xue F Qiao J Duan J Chen F. Evaluation of salivary biomarkers for the diagnosis of periodontitis. BMC Oral Hlth. (2021) 21:266. 10.1186/s12903-021-01600-5 34001101 PMC8130171

[21] Giannobile WV Beikler T Kinney JS Ramseier CA Morelli T Wong DT. Saliva as a diagnostic tool for periodontal disease: current state and future directions. Periodontol 2000. (2009) 50:52–64. 10.1111/j.1600-0757.2008.00288.x 19388953 PMC5695225

[22] Pourgonabadi S Müller HD Mendes JR Gruber R. Saliva initiates the formation of pro-inflammatory macrophages in vitro. Arch Oral Biol. (2017) 73:295–301. 10.1016/j.archoralbio.2016.10.012 27825074

[23] Müller H-D Cvikl B Lussi A Gruber R. Salivary pellets induce a pro-inflammatory response involving the TLR4-NF-kB pathway in gingival fibroblasts. BMC Oral Hlth. (2016) 17:15. 10.1186/s12903-016-0229-5 27430277 PMC4948095

[24] Müller HD Cvikl B Lussi A Gruber R. Chemokine expression of oral fibroblasts and epithelial cells in response to artificial saliva. Clin Oral Invest. (2016) 20:1035–42. 10.1007/s00784-015-1582-5 26342602

[25] Cvikl B Lussi A Moritz A Sculean A Gruber R. Sterile-filtered saliva is a strong inducer of IL-6 and IL-8 in oral fibroblasts. Clin Oral Invest. (2015) 19:385–99. 10.1007/s00784-014-1232-3 25115993

[26] Gröschl M Topf HG Kratzsch J Dötsch J Rascher W Rauh M. Salivary leptin induces increased expression of growth factors in oral keratinocytes. J Mol Endocrinol. (2005) 34:353–66. 10.1677/jme.1.01658 15821102

[27] Caton JG Armitage G Berglundh T Chapple ILC Jepsen S Kornman KS. A new classification scheme for periodontal and peri-implant diseases and conditions - introduction and key changes from the 1999 classification. J Periodontol. (2018) 89:S1–8. 10.1002/JPER.18-0157 29926946

[28] Navazesh M Kumar SKS. Measuring salivary flow: challenges and opportunities. J Am Dent Ass. (2008) 139:35S−40. 10.14219/jada.archive.2008.0353 18460678

[29] Gröger S Michel J Meyle J. Establishment and characterization of immortalized human gingival keratinocyte cell lines. J Periodontal Res. (2008) 43:604–14. 10.1111/j.1600-0765.2007.01019.x 18771458

[30] Ben Lagha A Grenier D. Black tea theaflavins attenuate *Porphyromonas gingivalis* virulence properties, modulate gingival keratinocyte tight junction integrity and exert anti-inflammatory activity. J Periodontal Res. (2017) 52:458–70. 10.1111/jre.12411 27549582

[31] Gilchrist EP Moyer MP Shillitoe EJ Clare N Murrah VA. Establishment of a human polyclonal oral epithelial cell line. Oral Surg Oral Med Oral Pathol Oral Radiol. (2000) 90:340–7. 10.1067/moe.2000.107360 10982956

[32] Feghali K Tanabe S Grenier D. Soluble CD14 induces cytokine release by human oral epithelial cells. J Periodontal Res. (2011) 46:147–52. 10.1111/j.1600-0765.2010.01311.x 21208208

[33] Kinane DF Preshaw PM Loos BG. Host-response: understanding the cellular and molecular mechanisms of host-microbial interactions - consensus of the seventh european workshop on periodontology. J Clin Periodontol. (2011) 38:44–8. 10.1111/j.1600-051X.2010.01682.x 21323703

[34] Reynolds JJ Meikle MC. Mechanisms of connective tissue matrix destruction in periodontitis. Periodontol 2000. (1997) 14:144–57. 10.1111/j.1600-0757.1997.tb00195.x 9567969

[35] Ramseier CA Kinney JS Herr AE Braun T Sugai JV Shelburne CA. Identification of pathogen and host-response markers correlated with periodontal disease. J Periodontol. (2009) 80:436446. 10.1902/jop.2009.080480 19254128 PMC5695217

[36] Ebersole JL Nagarajan R Akers D Miller CS. Targeted salivary biomarkers for discrimination of periodontal health and disease(s). Front Cell Infect Microbiol. (2015) 5:62. 10.3389/fcimb.2015.00062 26347856 PMC4541326

[37] Kawamoto D Amado PPL Albuquerque-Souza E Bueno MR Vale GC Saraiva L. Chemokines and cytokines profile in whole saliva of patients with periodontitis. Cytokine. (2020) 135:155197. 10.1016/j.cyto.2020.155197 32707521

[38] Groeger SE Meyle J. Epithelial barrier and oral bacterial infection. Periodontol 2000. (2015) 69:46–67. 10.1111/prd.12094 26252401

[39] Wang SS Tang YL Pang X Zheng M Tang YJ Liang XH. The maintenance of an oral epithelial barrier. Life Sci. (2019) 227:129–36. 10.1016/j.lfs.2019.04.029 31002922

[40] Ohshima M Sato M Ishikawa M Maeno M Otsuka K. Physiologic levels of epidermal growth factor in saliva stimulate cell migration of an oral epithelial cell line, HO-1-N-1. Eur J Oral Sci. (2002) 110:130–6. 10.1034/j.1600-0722.2002.11179.x 12013556

[41] Nighot P Al-Sadi R Rawat M Guo S Watterson DM Ma T. Matrix metalloproteinase 9-induced increase in intestinal epithelial tight junction permeability contributes to the severity of experimental DSS colitis. Am J Physiol Gastrointest Liver Physiol. (2015) 309:988–97. 10.1152/ajpgi.00256.2015 26514773 PMC4683300

[42] Komine KI Kuroishi T Ozawa A Komine Y Minami T Shimauchi H. Cleaved inflammatory lactoferrin peptides in parotid saliva of periodontitis patients. Mol Immunol. (2007) 44:1498–508. 10.1016/j.molimm.2006.09.003 17030385

[43] Bellagambi FG Lomonaco T Salvo P Vivaldi F Hangouët M Ghimenti S. Saliva sampling: Methods and devices. An overview. Trends Anal Chem. (2020) 124:115781. 10.1016/j.trac.2019.115781

